# Pro-environmental behavior, personality and emotional intelligence in adolescents: a systematic review

**DOI:** 10.3389/fpsyg.2024.1323098

**Published:** 2024-02-13

**Authors:** Paulo Vítor Lisboa, Cristina Gómez-Román, Lidia Guntín, Ana Paula Monteiro

**Affiliations:** ^1^CRETUS, Interdisciplinary Research Center in Environmental Technologies, University of Santiago de Compostela, Santiago de Compostela, Galicia, Spain; ^2^Department of Social Psychology, Basic Psychology and Methodology, Faculty of Psychology, University of Santiago de Compostela, Santiago de Compostela, Spain; ^3^Department of Education and Psychology, University of Trás-os-Montes and Alto Douro, Vila Real, Portugal; ^4^Centre for Educational Research and Intervention, University of Porto, Porto, Portugal

**Keywords:** pro-environmental behavior, adolescents, personality, emotional intelligence, systematic review

## Abstract

**Introduction:**

Human behavior significantly contributes to environmental problems, making the study of pro-environmental behavior an important task for psychology. In this context, it is crucial to understand the pro-environmental behavior of adolescents, as young people play a fundamental role in facilitating long-term changes in environmental consciousness and encouraging decision-makers to take action. However, little is currently known about the pro-environmental behavior of adolescents. Recently, there has been growing interest in examining the influence of personality traits and emotional intelligence on pro-environmental behavior.

**Methods:**

We conducted a systematic review to enhance our understanding of adolescent pro-environmental behavior. Thus, this systematic review was designed to enhance understanding of adolescent’s pro-environmental behavior by summarizing existing evidence on how it relates to personality and emotional intelligence.

**Results:**

Our findings suggest associations between specific personality traits and dimensions of emotional intelligence with adolescent pro-environmental behavior, aligning with similar studies conducted on adults.

**Discussion:**

While our findings offer valuable insights, further research is needed to establish causality and deepen our understanding of the interplay between multiple variables influencing pro-environmental behavior among adolescents.

**Systematic review registration:**

[https://www.crd.york.ac.uk/prospero/display_record.php?ID=CRD42023387836], identifier [CRD42023387836].

## 1 Introduction

Human behavior is considered one of the main factors responsible for environmental problems (Steg and de Groot, 2012; Cook et al., 2016; Vicedo-Cabrera et al., 2021). Therefore, pro-environmental behavior (PEB), defined as behavior that protects the environment (Stern, 2000; Kollmuss and Agyeman, 2002) or at least does not harm it (Lange and Dewitte, 2019), plays a key role in reducing environmental problems. For this reason—and to help define policies and programs that effectively promote PEB—much research has been conducted to identify the factors that influence people to take action (or not) to benefit the environment (for a review, see: Bamberg and Moser, 2007; Steg and Vlek, 2009; Gifford and Nilsson, 2014; Blankenberg and Alhusen, 2019). Considering that the severity of environmental problems keeps increasing (IPCC, 2023), this kind of research still needed.

The study of adolescents’ PEB is particularly important for the future of environmental protection (Balundė et al., 2020), as the behaviors of young people are key indicators of long-term changes in both environmental consciousness and action (Wray-Lake et al., 2010; Jovanović et al., 2016; Koessler et al., 2022). However, the nature of adolescents’ PEB is under-investigated (Palupi and Sawitri, 2018; Lee et al., 2020; Gong and Zheng, 2021).

The scientific literature has shown that PEB is determined by various factors. For example, Blankenberg and Alhusen (2019) present a review outlining 22 such factors, each categorized as socio-economic, psychological, habits, and contextual factors. The sheer number of factors highlights the difficulty of defining PEB-promotion policies, as numerous variables must be considered. Due to the multifaceted nature of PEB and this complex and evolving research landscape (Gifford and Nilsson, 2014), investigation has focused on identifying those factors that have the strong impact on PEB. In recent years, has gained relevance research about the role of variables such as gratitude (Sun et al., 2023), mindfulness (Panno et al., 2018; Apaolaza et al., 2022), connectedness to nature (see meta-analysis: Whitburn et al., 2020), use of smartphones (Fang et al., 2021), personality (e.g. Soutter and Mõttus, 2020; Gibbon and Douglas, 2021), or emotional intelligence (EI; e.g.: Aguilar-Luzón et al., 2014; Carrieri and Fermani, 2018).

This systematic review focuses on two of these variables: personality and EI. These constructs were chosen because psychological variables have made significant contributions to understanding of PEB (Li et al., 2019). Previous research has identified a link between PEB and some personality traits (for a meta-analysis, see Soutter et al., 2020), affect (Carrus et al., 2008; Coelho et al., 2017) and emotion (Durán et al., 2007; Robina-Ramírez et al., 2020), indicating that EI may be associated with PEB. In addition, these two variables develop substantially during adolescence. Research suggests that personality is continually maturing during adolescence (Van Dijk et al., 2020; Tetzner et al., 2023) and that an individual’s EI undergoes complex changes during this period, increasing in some dimensions and decreasing in others (Keefer et al., 2013; Azpiazu et al., 2022). Therefore, it is essential to explore how these two factors interact with adolescents’ PEB. Thus, the purpose of this systematic review is to summarize the available evidence on personality and EI are related to adolescents’ PEB, thereby making a valuable Contribution To The Field of environmental psychology.

### 1.1 Why adolescents?

The research into children’s environmental attitudes and behavior began some decades ago (Otto et al., 2019), with the goal of clarifying the origins and development of both ecological sense and environmental behavior. Hahn and Garrett (2017) demonstrated that children of 3 years were already able to evaluate actions as environmentally harmful, showing moral attitudes. In a more recent study, Geraci et al. (2023) investigated the environmental morality of 7-month-old infants. The authors highlight the results of well-established studies indicating that young children make moral judgments of actions that harm the environment and seek to clarify when this moralization begins. Of importance to the current study, their results show that, by the age of 7 months, children are able to make moral evaluations. This may suggest that PEB can be part of an innate propensity (Otto et al., 2021; Geraci et al., 2023), crucial for environmental protection. Indeed, empirical evidence suggests that by the time a child has reached the age of 7 years their attitude and behavior have formed (Otto et al., 2019).

In a study of adolescents’ PEB, Böhme et al. (2018) found that adolescents seem to be potentially influenced, in the case by mindfulness, to actively engage in sustainable consumption. Collado et al. (2019) report that young people influence one another to environmentalist behavior. Similarly, Žukauskienė et al. (2021) conclude that adolescents may be important agents to influence their families and communities to adopt pro-environmental attitudes and PEB. We have witnessed the widespread participation of teenagers in the “Fridays for the Future” initiative, a movement initiated by Greta Thunberg, which may indicate greater concern with environmental issues.

Taken together, these results are a positive signs for the environmental-protection cause, as it seems that—from as young as 7 months old and throughout infant–juvenile development—young people may engage in environmental protection as a development task. However, research also indicates a decline in PEB during adolescence (Collado et al., 2015; Krettenauer, 2017; Wray-Lake et al., 2017; Krettenauer et al., 2019; Otto et al., 2019; Keith et al., 2021). Additionally, a discrepancy between environmental attitudes and behaviors has been observed in adolescents, with their concerns not always translating into corresponding behaviors (Huoponen, 2023; Thomaes et al., 2023). Therefore, it remains unclear whether adolescents’ involvement in environmental issues indicates a genuine commitment to environmental protection or whether it is a demand for governmental action rather than an assertion of individual responsibility (Wray-Lake et al., 2010). Given these mixed results, further investigation is vital to enhance our understanding of the factors driving adolescents’ PEB.

Although we are aware of the view that adolescence should be defined by the age range 10–24 years (e.g., Sawyer et al., 2018), our study focuses on adolescents aged between 11 and 18 years. This decision was taken because, in several domains of psychology, investigations of adolescents focus on this age range (e.g., Böhme et al., 2018; Gómez-López et al., 2019; Balundė et al., 2020; Habib et al., 2023; Neurohr et al., 2023). Furthermore, we argue that extending the age range to 24 years could make comparison and comprehension of the behaviors under study more difficult, as it would introduce greater variability into the population under study (e.g., by including university students and participants already in the job market).

### 1.2 Personality and pro-environmental behavior

Personality traits play a fundamental role in shaping an individual’s beliefs, attitudes, and values, which, in turn, influence their behaviors and decisions (Markowitz et al., 2012; Soutter et al., 2020). On that basis, there is a growing body of research into the relationship between personality and PEB (Soutter and Mõttus, 2020). These studies tend to apply the “five-factor model of personality,” which comprises the so-called “Big Five”: Extraversion, Agreeableness, Conscientiousness, Neuroticism, and Openness to experience. Alternatively, some use the HEXACO model and its six dimensions: Honesty–Humility, Emotionality, Extraversion, Agreeableness, Conscientiousness, and Openness to Experience. Some researchers have applied the NEO Personality Inventory (Costa and McCrae, 1992), in one of its several versions (e.g., Zhou et al., 2019), while others have employed several versions of the HEXACO–Personality Inventory–Revised (Lee and Ashton, 2004; Ashton and Lee, 2009; Brick and Lewis, 2016; Pavalache-Ilie and Cazan, 2018; Panno et al., 2021) or the International Personality Item Pool (Johnson, 2014; Soutter and Mõttus, 2020). These studies usually examine the relationships between personality and PEB, pro-environmental attitude (PEA) or pro-environmental intention (PEI).

Soutter et al. (2020) conducted a meta-analysis of investigations in which the “Big Five” or HEXACO models had been used to examine the relationship between personality, PEB, and PEA. Their results show that Openness, Honesty–Humility, Agreeableness, Conscientiousness, and Extraversion are associated with both PEA and PEB, with Openness and Honesty–Humility having the strongest correlations. Panno et al. (2021) also found that Openness and Honesty–Humility were significant predictors of PEB, and these traits were linked to PEB both directly and indirectly through moral anger. Similarly, research by Markowitz et al. (2012) identified a moderate positive relationship between Openness and PEB, while Brick and Lewis (2016) found that Openness and Conscientiousness independently predict emission-reduction behavior and that their effects are mediated by PEA. Openness was also found to be correlated with PEB in a study by Puech et al. (2019), while Gibbon and Douglas (2021) found that Openness/Intellect significantly predict PEB. Analyzing personality facets, Soutter and Mõttus (2020) found that Openness, Agreeableness, and Conscientiousness—along with certain aspects of Extraversion—positively predicted both PEA and PEB, while certain facets of Neuroticism had a negative association with PEB. An investigation among village leaders in China found that leaders with higher levels of Agreeableness and Neuroticism were more willing to adopt environmental protection measures (Zhou et al., 2019). These findings suggest that personality may be one explanation for engagement in PEB during adolescence.

Of special interest to the current systematic review is the development of this propensity to environmental protection, as well the possibility that one indicator of this propensity may be the personality trait of Honesty–Humility (Otto et al., 2021). Moreover, although personality develops across the lifespan, adolescence is a period that sees major development of the personality (Soto et al., 2011; de Moor et al., 2022), as well as its maturation (McCrae et al., 2002; Van Dijk et al., 2020). In addition, mean levels of Agreeableness, Conscientiousness, and Openness generally rise during adolescence (Soto et al., 2011; Van Dijk et al., 2020). As a result, it would be beneficial to clarify the extent to which personality influences PEB during that period.

### 1.3 Emotional intelligence and pro-environmental behavior

In the quest to investigate factors that influence PEB, researchers have explored the crucial role of emotions. In this context, EI is a promising variable. Research in this domain has used the Wong and Law (2002) Emotional Intelligence Scale (e.g., Aziz et al., 2021); the Salovey et al. (1995) Trait Meta-Mood Scale (e.g., Aguilar-Luzón et al., 2014); and The Trait Emotional Intelligence Questionnaire (Petrides, 2009a,b) in its various forms (e.g., Ntanos et al., 2017; Giancola et al., 2022). Aguilar-Luzón et al. (2014) studied the role of EI as a moderating variable in the relationship between anthropocentric beliefs, ecocentric beliefs, and PEB. Their results show that those people with stronger ecocentric beliefs and a better ability to manage their emotions tend to have more favorable attitude toward behavior, greater intention to have PEB and more engagement in PEB. Furthermore, when anthropocentric beliefs are less pronounced and EI is higher, there is a greater intention to perform PEB. Chowdhury (2017), in a study of the relationship between EI and ethical consumption, found that an ability to appraise and recognize other’s emotions was positively related to pro-environmental buying actions. Ntanos et al. (2017) found a positive correlation between trait EI and willingness to invest in renewable energy sources. Likewise, Carrieri and Fermani (2018) show a positive correlation between EI and sustainable-hospitality choices through social well-being, and using a cluster analysis, they found that higher scores for IE correspond to a stronger sustainable-hospitality orientation. Further, Aziz et al. (2021) found that EI mediated the relationship between pro-environmental intention and PEB. Together, these results suggest that the role of EI in the understanding and promotion of PEB warrants further study.

### 1.4 Strategic question

Although there is growing evidence of relationships between personality and PEB and between EI and PEB, there are gaps in this knowledge—namely, regarding the role of the variables in the PEB of adolescents. The present research examines the studies in this field and considers their conclusions in relation to adolescents to gain new insights. The findings of the current paper could support the design of PEB-promotion programs catered to specified personality traits and aspects of EI.

## 2 Methods

### 2.1 Databases and search strategy

Searches were conducted in seven electronic databases: Proquest, which included PsycARTICLES, ERIC and Psychology Database; MEDLINE; PubMed; Scopus; and Web of Science. In each database, the following combinations of keywords was searched: (Adolesc* OR “Young people” OR Youth* OR Teen*) AND (“Proenvironmental behavior” OR “pro-environmental behavior” OR “ecological behavior” OR “sustainable behavior” OR “environmentally friendly behavior” OR “green behavior”) AND (Personality OR “big five” OR “five factor model” OR extravers* OR neurotic* OR “emotional stability” OR openness OR agreeableness OR conscientiousness) AND (“Emotional Intelligence” OR “Self-Emotion Appraisal” OR “Others’ Emotion Appraisal” OR “Use of Emotion” OR “Regulation of Emotion”).

### 2.2 Screening and detailed assessment process

The Preferred Reporting Items for Systematic Reviews and Meta-analyses (PRISMA) guidelines for systematic reviews were followed (Page et al., 2021) and the systematic review protocol was registered through the PROSPERO—International Prospective Register of Systematic Reviews (CRD42023387836).

**FIGURE 1:**
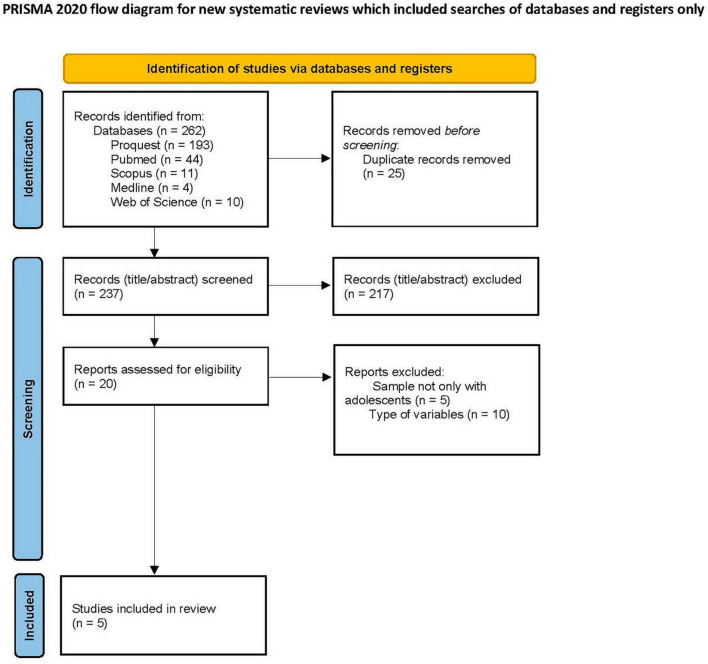
PRISMA diagram, which collects the different steps followed in the process for the final selection of studies included in the systematic review.

Papers were downloaded from a Mendeley library. A search of the databases yielded a total of 262 records (Figure 1), and 25 duplicates were removed from these. Researchers, independently and simultaneously, screened the titles and abstracts of the remaining 217 articles. The following inclusion and exclusion criteria were applied throughout the screening process. All discrepancies were discussed to reach final decisions based on consensus among the evaluators.

### 2.3 Inclusion and exclusion criteria

To select the articles for the review, the following inclusion criteria were used: (1) only peer-reviewed and empirical articles in full-text form were considered acceptable. All articles were published in English, Spanish, or Portuguese. All dates were acceptable. (2) The participants in the studies were to be adolescents aged 11–18 years. (3) The studies must have applied quantitative, qualitative, or mixed methods, but in the case of meta data, only quantitative methods—such as meta-analysis—would be analyzed.

The reasons for excluding an article were as follows: (1) it was not available in full text; (2) it was published in a language other than English, Spanish, or Portuguese; (3) it did not use a defined tool to directly measure PEB, personality, or emotional intelligence; and (4) it was a review, opinion, editorial, case study, or empirical study in which the relationship between PEB and personality or between PEB and emotional intelligence were merely mentioned or superficially discussed, without in-depth analysis.

### 2.4 Final selection process

On the basis of this initial screening of the abstracts, 20 articles were deemed relevant for further assessment. All 20 articles were screened at the full-text stage by two researchers who chose the papers for final inclusion. All of the uncertainties (i.e., five highlighted by one researcher and nine by the other) were discussed with the two other researchers, and agreement was reached on the five articles included in this systematic review. (For the reasons for the exclusions, see Figure 1).

### 2.5 Quality and risk bias of selected studies

The methodological quality of the included studies was assessed using an Excel spreadsheet that listed all of the studies, duly coded. Extraction of relevant information from the selected primary studies was performed in the referred excel spreadsheet. Specifically, final analysis consisted in verifying two items: (1) whether the sample included only adolescents and (2) whether the variables under study included PEB, personality, or emotional intelligence. The two evaluators discussed their respective analyses, and once all four researchers had discussed the disagreements, an agreement was reached on the final decision.

## 3 Results

This systematic review included a total of five studies, and a summary of the main findings can be found in Table 1. The small number of articles included in the final selection reflects the scarcity of the research on these topics within the literature on PEB. All the included studies were published in the English language and had been conducted between 2014 and 2020. Although the studies analyzed the relationships between several variables and PEB, only those results concerning PEB, PEA, or PEI will be reported in this review. Despite our primary focus on PEB, we consider it worthwhile to include data on PEA and PEI because one of the most widely applied theories in PEB studies—the Theory of Planned Behavior (Ajzen, 1991)—postulates that attitude directly influences intention, which in turn leads to the behavior. Although research shows that attitude not always turns in behavior, inconsistency known as the “attitude–behavior gap” and studied extensively in the field of environmental behavior (Siegel et al., 2018)—evidence also shows that intention is the strongest determinant of behavior (Klöckner, 2013).

**TABLE 1:** Characterization of articles selected for analysis of the systematic literature review (*n* = 5).

References	Country	Sample	Age range/Mage	Research objectives	Design	Measures of interest to this systematic review	Main findings
Quintelier (2014).	Belgium	*N* = 3.426 Female: 54% Male: 46%	15 years old Mage = 15	Use the “Big Five” personality structure to explore the relationship with political consumer behavior.	Cross sectional	- 3 bipolar items that assessed each personality trait - 1 single item that assessed intention to boycott - 12 items that assessed environmental concern	- Openness to experience leads to more political consumer behavior. - Agreeableness or trust in people also often has a positive effect on intention to boycott. - The effects of extraversion tend to be negative.
Poškus and Žukauskienė (2017)	Lithuania	*N* = 612 Female: 42% Male: 58%	13–17 Mage = 15.25	Investigate whether adolescents with specific configurations of personality traits approach recycling differently and whether or not their perceived behavioral control as well as their attitudes and personal norms regarding recycling differ among cluster.	Cross sectional	- 44-items The Big Five Inventory−BFI - 1 item that assessed recycling intention - 1 items that assessed self-reported behavior	- Adolescents that have different personality types approach recycling differently. - Those who have more expressed adaptive and positive personality traits show more favorable attitudes toward recycling and engage in recycling more than those who have less adaptive traits.
Robinson et al. (2019)	Australia	*N* = 406 Female: 24% Male: 76%	12–17 Mage = 14.48	Investigate the relationship between self-reported EI, PEA and PEB.	- Cross sectional	- Pro-environmental Behaviors Scale (PEBS) - 57-item Adolescent Swinburne University Emotional Intelligence Teste (SUEIT)	- Higher PEB was associated with EI dimensions of Emotional Management and Control (EMC) and Understanding Others Emotions (UEO). - Hierarchical multiple regression confirmed that EMC also predicted PEB. - EMC and UEO interactively moderated the relationship between PEA and PEB.
Poškus (2020a)	Lithuania	*N* = 863 Female: 54% Male: 46%	n.a Mage = n.a	Understanding whether individuals who have different patterns of personality traits react differently to persuasive messages that are tailored to promote PEB.	- Experimental	- 44 items The Big Five Inventory−BFI - 1 item that assessed Pro-environmental intention for each behavior	- Adolescents with different patterns of personality traits are differently affected by persuasive messages. - Adolescents with different patterns of personality traits in general approach PEB differently.
Poškus (2020b)	Lithuania	*N* = 863 Female: 53.5% Male: 46.5%	14–18 Mage = 15.72	Exploring the moderating effect of personality profiles on behaviors.	- Cross sectional	- 44 items The Big Five Inventory−BFI - 1 item that assessed intention to behave for each behavior - 1 item that assessed self-reported behavior for each behavior	- Individuals with different patterns of personality traits approach PEB differently.

An assessment of the methodologies revealed that the variables had been assessed using self-reported measures. There were a total of 6,170 participants across the studies, with an average of 1,234 per study. In one study, the age of the participants was not specified, while the other four studies focused on adolescents with an average age of 15.11 years (ranging from 12 to 18 years). In one study, only 24% of the participants were female, whereas the remaining four studies had gender-balanced samples, with an average across the samples of 48.87% female (ranging from 42 to 54%). Regarding the geographic context, one study was conducted in Australia and the others in Europe.

### 3.1 Evidence of the effect of personality on adolescents’ PEB

One aim of the current systematic review was to examine the role of personality in influencing adolescents’ PEB. Four of the five studies evaluated this. One study (Quintelier, 2014) used the “Big Five” personality model to examine the correlation between personality and political consumer behavior, specifically the intention to boycott products for social, political, or environmental reasons. It found a correlation between Openness to experience and political consumption behaviors. A regression analyses showed that Openness has a positive effect on the intent to boycott, while Extraversion has a negative effect: that is, participants who are open-minded, imaginative, curious, creative, and insightful (McCrae and John, 1992) are more likely to boycott products for social, political, and environmental reasons. In contrast, participants with characteristics of sociability, talkativeness, assertiveness, and excitability (McCrae and John, 1992) are less likely to boycott.

Another study (Poškus and Žukauskienė, 2017) focused on adolescents’ recycling behavior. Taking a person-oriented approach, the study was designed to verify not only the relationship between recycling behavior and personality traits, but also to cluster the participants into groups of similar people using the “Big Five” personality traits. Specifically, the goal was to investigate how different configurations of personality traits influenced, among other variables, attitude, intention, behavior. The first analyses found that Agreeableness and Conscientiousness have moderate correlations with recycling attitudes and intention, while Agreeableness is also related to self-reported recycling behavior. Further analysis indicates four clusters: (1) “Positive,” which includes adolescents who score highly for Extraversion, Agreeableness, Conscientiousness, Openness, and Neuroticism; (2) “Negative,” which includes those with high Neuroticism and low Extraversion, Agreeableness, Conscientiousness, and Openness; (3) “Extravert and Open,” which includes adolescents with high scores for Extraversion and Openness, moderate scores for Conscientiousness and Neuroticism, and low scores for Agreeableness; and (4) “Agreeable and Closed groups,” which include those with high scores for Agreeableness and low for Openness. Adolescents in the positive cluster reported more recycling behavior than those in the other three clusters, although this difference was only marginal. In addition, the adolescents in this cluster differed significantly from the others in terms of their intention to recycle and attitudes toward recycling.

In another sample, Poškus (2020a), using the same person-oriented approach, grouped the adolescents into personality-trait clusters to study the effect of personality on five pro-environmental intentions and behaviors: recycling, water conservation, electricity conservation, sustainable consumption, and sustainable-transportation use. The four final clusters differed slightly from those in the previous study (Poškus and Žukauskienė, 2017): (1) “Positive,” grouping individuals who are stable, social, friendly, responsible, and open to new ideas and experiences; (2) “Conservative,” whose members are low in Openness and relatively emotionally stable; (3) “Outgoing,” grouping adolescents who score highly for Extraversion; and (4) “Negative,” comprising individuals who are moderately disagreeable and unconscientious, without refusing novel ideas. The results reveal that the Positive cluster displayed the highest pro-environmental scores, while the Conservative cluster had the lowest. The Negative and Outgoing clusters received average scores for their pro-environmental tendencies, with the Negative cluster being slightly more open to PEB.

Finally, in another study (Poškus, 2020b) examined the association between personality and reaction to persuasive messages encouraging pro-environmental behavior such as recycling and the conservation of water and electricity. This was a large intervention study, predating the previously summarized work (Poškus, 2020a), and it included the same clusters of participants. However, the goal of this research was to verify whether people with different personality patterns were differently affected by persuasive messages in terms of the effects on their respective PEI and PEA. At the starting point, the Positive cluster exhibited the highest level of PEB, while the Conservative cluster displayed the lowest, and the Negative and Outgoing clusters in the range between. After the intervention, the Positive cluster continued to have higher scores, being more influenced by the persuasive messages. Conversely, the Negative cluster was least affected by the persuasive messages. Interestingly, participants in the Conservative cluster, despite their characteristic resistance to adopting new behaviors, did evidence some changes. The strongest effect was seen for the Outgoing cluster, meaning that those adolescents who are more reactant to salient social norms will react more strongly to interventions that promote pro-environmental behavior as a salient social norm, which has been done in this study.

### 3.2 Evidence on the effect of emotional intelligence on adolescents’ pro-environmental behavior

The second aim of this review is to examine the relationship between EI and PEB during adolescence. A search for studies of this relationship found just one article (Robinson et al., 2019). That study involved the Adolescent Swinburne University Emotional Intelligence Test, which measures four EI traits: (1) Emotional Recognition and Expression (ERE), (2) Understand Emotions of Others (UEO); (3) Emotions Direct Cognition (EDC); and (4) Emotional Management and Control (EMC). The aim was to evaluate whether EI was associated with PEB and PEA and whether the relationship between PEA and PEB was moderated by EI.

The correlation analyses in this study revealed a moderate positive relationship between PEA and PEB, indicating that stronger pro-environmental attitudes are related to higher levels of pro-environmental behavior. Conversely, EMC is inversely correlated with PEA, suggesting that individuals with better emotional management and control may have weaker pro-environmental attitudes. Concerning the potential moderating effect of EI on the relationship between PEA and PEB, the study found that only UEO and EMC had the potential to act as moderators, occurring when both UEO and EMC are low or high. A hierarchical multiple regression confirmed that EI explains an additional 3% of the variance in the model and that higher EMC predicts higher PEB. The results confirm the presence of moderation, suggesting that these dimensions of EI—in particular EMC—play a role in influencing the relationship between pro-environmental attitudes and behavior.

Overall, this study provides evidence that emotional intelligence—particularly the dimension of emotional management and control—is associated with pro-environmental behavior during adolescence. It also suggests that certain aspects of emotional intelligence may moderate the relationship between PEA and behavior.

## 4 Discussion

The commitment of the younger generation to PEB is vital for environmental protection, so it is equally important to identify the factors that could increase that commitment. In recent years, various studies have focused on the psychological variables contributing to this, although investigations with adolescents remain rare. This systematic review synthesized the evidence related to adolescents’ PEB and the association with both personality and emotional intelligence. To our knowledge, no other systematic reviews have specifically addressed these concepts in the context of adolescent PEB. In summary, the main findings of this review are as follows: (1) personality and EI traits can be empirically distinguished in adolescence, (2) certain personality traits or clusters and specific dimensions of EI are more strongly associated with environmentalism than others, and (3) the data could underpin suggestions for promoting the development of PEB in adolescence.

Although the results indicate a relationship between adolescents’ personality, EI, and PEB, it is important to note that the current literature does not establish a causal relationship. Only one of the five studies included in this review employed an experimental design, while the others were correlational in nature. Therefore, more research is needed to deepen our understanding in this area. As noted previously, a range of variables is associated with PEB and it may be interesting to research the interplay between those variables and personality and EI. Nevertheless, the results of this review indicate that these findings from studies with adolescent samples are consistent with those from studies of adults.

Agreeableness and Openness to experience were shown by Quintelier (2014) to lead to more political consumption, and the same “positive” cluster is also more pro-environmental (Poškus and Žukauskienė, 2017; Poškus, 2020a,b). These results are consistent with those for adult samples (Markowitz et al., 2012; Brick and Lewis, 2016; Puech et al., 2019; Soutter and Mõttus, 2020; Gibbon and Douglas, 2021; Panno et al., 2021). Furthermore, Poškus (2020b) shows that individuals in the Positive cluster, after exposure to persuasive messages, become even more environmentalist. This could mean that treating PEB as something new may increase PEB, even in those who are already pro-environmentalist. Quintelier (2014) concludes that the extraversion trait has a negative effect on the political consumption. Conversely, the clusters “Extravert and Open” (Poškus and Žukauskienė, 2017) and “Outgoing” (Poškus, 2020a,b)—which comprise those with high scores for Extraversion—are associated with average levels of PEB. This might suggest that the associations between personality and PEB could be clarified by research that—rather than analyzing traits—takes a person-oriented approach, grouping individuals holistically and according to the patterns between them (Poškus, 2018). This suggestion becomes more significant when considering the research by Poškus (2020b) concerning the “Conservative” cluster. Being characterized by low openness, average neuroticism, and average scores in other traits, these individuals tend to be resistant to novel ideas and values—attributes required for the adoption of PEB (Poškus, 2018). However, surprisingly, interventions have shown that even individuals in this cluster can experience improvements in their PEB levels in response to pro-PEB messaging. This highlights the potential of to promote PEB among all individuals, regardless of their personality traits. Different personality clusters react differently to persuasive messaging, and these results suggest that personalizing messages to align with different traits or personality profiles could make them more effective. Indeed, previous research has shown that a message’s persuasiveness is influenced by the personality of the recipient (Pangbourne et al., 2020) and that personalized messages are likely to be more successful than generic messaging (Jylhä et al., 2013). Empirical evidence shows that personality traits can change (Jach et al., 2023)—throughout the lifespan or in response to interventions. In adolescence, these changes are very pronounced (Bleidorn et al., 2018). Therefore, the need for personality-change interventions and personalized learning (Jach et al., 2023) may be another practical implication of these results. The idea is to combine environmental science with behavioral change to designing interventions to promote, for example, the traits needed to become more open to the novelty of PEBs or less resistant to their costs (Niu et al., 2023) and that usually prevent it.

As noted earlier, the dimensions of Emotional Intelligence most strongly correlated with PEB in the sample of adolescents are “Emotions Management and Control” and “Understanding Emotions of Others.” Although this correlation is moderate, there is evidence—from both the adult samples (Mattingly and Kraiger, 2019) and the children and adolescent samples (Durlak et al., 2011)—that EI can be enhanced with training. Given this promising connection, it could be beneficial to incorporate EI development into environmental education programs. By linking sustainability with emotional skills, we can strengthen the relationship between pro-environmental behavior and emotional intelligence. The data obtained from this systematic review provides support for the claim that personality and EI are related to PEB, and furthermore, these findings could contribute to environmental-education initiatives, PEB programs, and interventions to deal with eco-anxiety.

EI includes the ability to perceive, understand, monitor, and regulate one’s own and others’ feelings and to use this information to guide one’s own actions. In the context of this study, a plausible hypothesis is that the ability to manage the emotions associated with environmental protection will allow a person to engage in mechanisms of adaption, such as PEB. For example, approaches to managing eco-anxiety include the provision of emotional support and emotional-focused interventions. (For a scoping review, see Baudon and Jachens, 2021). This assigns to EI the important role of not only intervening to mitigate the negative impact of environmental problems on mental health, but also linking EI and the ability to take action for the environmental protection. Future research might examine this potentiality by creating environmental education programs that acknowledge this link.

## 5 Limitations and strengths

The small number of studies (*n* = 5) included in this review is the greatest limitation of this work. To overcome this, future studies could include both peer-reviewed journals and so-called “gray literature.” Still regarding the number of studies, we are aware that when this happens a meta-analyses could be considered. However, that was not possible in this case, as the studies were too heterogeneous to be comparable (Lee, 2019) and the data was not sufficient to permit generalization (Allen, 2020). Nevertheless, this limitation might reflect the under-researched nature of this topic, explained by the novelty of the area of study or the focus on these particular variables —aspects which could also be considered strengths. To measure both personality traits and PEB, a plurality of instruments was used, which may also be a limitation. Although all the studies in the review use the “Big Five” model, Quintelier (2014) used a different scale to Poškus and Žukauskienė (2017), Poškus (2020a), and Poškus (2020b). In the same way, the study by Robinson et al. (2019) applied a scale to measure PEB, but in all the other studies, no specific instruments were used to measure PEB.

Despite these limitations, the goal and findings of this review can be considered strengths, as this is—to the best of our knowledge—the first systematic review to consider the relationship between adolescents’ personality, emotional intelligence, and pro-environmental behaviors and how this influences the processes that drive adolescents’ PEB.

## 6 Conclusion

In conclusion, the findings of this review demonstrate the need for further research on the relationship between personality, emotional intelligence, and PEB in adolescents. This review suggests that certain personality traits and dimensions of emotional intelligence are associated with PEB, affecting adolescents in a manner consistent with that shown by previous studies to affect adults. This has important implications for research and practice, including for policymakers and/or educational programs’ designers to how to design interventions to promote PEB in different traits or clusters of personality and EI. However, more experimental studies and an exploration of the interplay between multiple variables are necessary to establish causality and deepen our understanding of these relationships in the context of adolescent PEB.

## Data availability statement

The original contributions presented in this study are included in this article/supplementary material, further inquiries can be directed to the corresponding author.

## Author contributions

PL: Conceptualization. Writing−original draft, Data curation, Formal Analysis, Investigation, Methodology. CG-R: Conceptualization, Formal Analysis, Investigation, Methodology, Supervision, Validation, Writing−review and editing. LG: Formal Analysis, Investigation, Methodology, Writing−review and editing. AM: Conceptualization, Formal Analysis, Investigation, Validation, Writing−review and editing.
